# Characterization of Two Toxin-Antitoxin Systems in Deep-Sea *Streptomyces* sp. SCSIO 02999

**DOI:** 10.3390/md17040211

**Published:** 2019-04-04

**Authors:** Waner Zhan, Jianyun Yao, Kaihao Tang, Yangmei Li, Yunxue Guo, Xiaoxue Wang

**Affiliations:** 1 Key Laboratory of Tropical Marine Bio-resources and Ecology, Guangdong Key Laboratory of Marine Materia Medica, RNAM Center for Marine Microbiology, South China Sea Institute of Oceanology, Chinese Academy of Sciences, Guangzhou 510301, China; zhanwaner16@mails.ucas.ac.cn (W.Z.); jianyunyao@scsio.ac.cn (J.Y.); khtang@scsio.ac.cn (K.T.); liyangmei13@mails.ucas.ac.cn (Y.L.); 2 University of Chinese Academy of Sciences, Beijing 100049, China

**Keywords:** toxin-antitoxin system, YoeB/YefM, deep sea, *Streptomyces*

## Abstract

Toxin-antitoxin (TA) systems are ubiquitous and abundant genetic elements in bacteria and archaea. Most previous TA studies have focused on commensal and pathogenic bacteria, but have rarely focused on marine bacteria, especially those isolated from the deep sea. Here, we identified and characterized three putative TA pairs in the deep-sea-derived *Streptomyces* sp. strain SCSIO 02999. Our results showed that Orf5461/Orf5462 and Orf2769/Orf2770 are *bona fide* TA pairs. We provide several lines of evidence to demonstrate that Orf5461 and Orf5462 constitute a type-II TA pair that are homologous to the YoeB/YefM TA pair from *Escherichia coli*. Although YoeB from SCSIO 02999 was toxic to an *E. coli* host, the homologous YefM antitoxin from SCSIO 02999 did not neutralize the toxic effect of YoeB from *E. coli*. For the Orf2769/Orf2770 TA pair, Orf2769 overexpression caused significant cell elongation and could lead to cell death in *E. coli,* and the neighboring Orf2770 could neutralize the toxic effect of Orf2769. However, no homologous toxin or antitoxin was found for this pair, and no direct interaction was found between Orf2769 and Orf2770. These results suggest that Orf2769 and Orf2770 may constitute a novel TA pair. Thus, deep-sea bacteria harbor typical and novel TA pairs. The biochemical and physiological functions of different TAs in deep-sea bacteria warrant further investigation.

## 1. Introduction

Toxin-antitoxin (TA) systems are ubiquitous genetic elements that consist of a toxin and an antitoxin that specifically neutralize the cognate toxin [1,2]. Based on the nature of the antitoxin and the mode of interaction, TA systems have been broadly divided into six types (types I-VI) [3,4]. Previous work on TA systems has mostly focused on *E. coli* strains and pathogens. To date, more than 37 TA systems have been identified in *E. coli* K-12 MG1655 [1]. TA systems have also been identified in some major pathogens, such as *Streptococcus pneumonia* [5], *Mycobacterium tuberculosis* [6], *Salmonella* [7], and *Pseudomonas aeruginosa* [8,9,10]. Although most of the TAs are well studied in *E. coli*, TAs with new functions and characteristics have recently been discovered in other microorganisms. For example, the type-III TAs ToxI/ToxN were first characterized in the *Erwinia carotovora* subspecies *atroseptica* [11] and the toxin ToxN acts as an endoribonuclease [12]. In addition, the newly named type-VI TA SocB/SocA system was identified in *Caulobacter crescentus*, and the toxin SocB can interact with the sliding clamp (driving replication fork collapse) to block replication elongation [13]. The novel toxin ParS of the ParS/ParT TA pair, a mono-ADP-ribosyltransferase (mART), specifically modifies phosphoribosyl pyrophosphate synthetase in soil bacterium *Sphingobium* sp. [14]. The novel toxin DarT of the DarT/DarG TA pair from the pathogen *Mycobacterium tuberculosis* causes reversible DNA ADP-ribosylation [15]. Furthermore, the toxin AtaT of the AtaT/AtaR TA pair from enterohemorrhagic *E. coli* blocks translation initiation by *N*-acetylation of the initiator tRNA^fMet^ [16,17]. TA systems have been found to be widely distributed in microorganisms of freshwater and marine sources using bioinformatics analysis, and some of these TAs have been verified and studied. For example, seven TAs in the estuarine *Synechococcus* strain [18] and three TAs, including HipA/HipB, HEPN/MNT and ParEso/CopAso, in *Shewanella oneidensis* isolated from Lake Oneida (NY) [19,20,21] were identified and characterized recently. Although the marine ecosystem harbors the largest quantities and greatest diversity of microorganisms [22], the TAs in marine bacteria are much less well explored. The type-II TA pair VapC/VapB from a deep-sea *Streptomyces* sp. has been verified [23] and the antitoxin ToxN (cognate toxin ToxI) in the marine bacterium *Vibrio harveyi* [11] and toxin PndA (homologous to the toxin Hok) in the marine pathogen *Vibrio parahaemolyticus* [24] have been predicted. Thus, new types of TA systems or toxins with novel functions remain to be identified in extreme marine environments.

*Streptomyces* sp. SCSIO 02999 (hereafter referred to as SCSIO 02999) was isolated from South China Sea sediment at a depth of 880 m and is taxonomically close to *Streptomyces* sp. VTT E-062988, ACT-40 and 1A01691 [25]. SCSIO 02999 is a Gram-positive bacterium and can produce multifarious biologically active compounds with antiviral, antitumor or antibacterial activity [26,27,28,29]. We have previously characterized a type-II TA pair VapC/VapB in SCSIO 02999 [23]. In this study, we analyzed three putative TA pairs in SCSIO 02999, Orf5461/Orf5462, Orf2769/Orf2770, and Orf2767/Orf2766 and showed that Orf5461/Orf5462 and Orf2769/Orf2770 are *bona fide* TA pairs. We demonstrated that Orf5461/Orf5462 is homologous to the type-II YoeB/YefM TA pair in *E. coli*. Overexpressing YoeB from SCSIO 02999 was toxic to an *E. coli* host, but the antitoxin YefM from SCSIO 02999 could not neutralize the toxic effect of YoeB from *E. coli*. Orf2769 and Orf2770 may constitute a novel TA pair, and the overexpression of Orf2769 led to the formation of elongated *E. coli* cells. Further studies are needed to explore the physiological functions of different TA systems in deep-sea bacteria.

## 2. Results

### 2.1. Identification of Two TA Pairs in Streptomyces sp. SCSIO 02999

**Figure 1:**
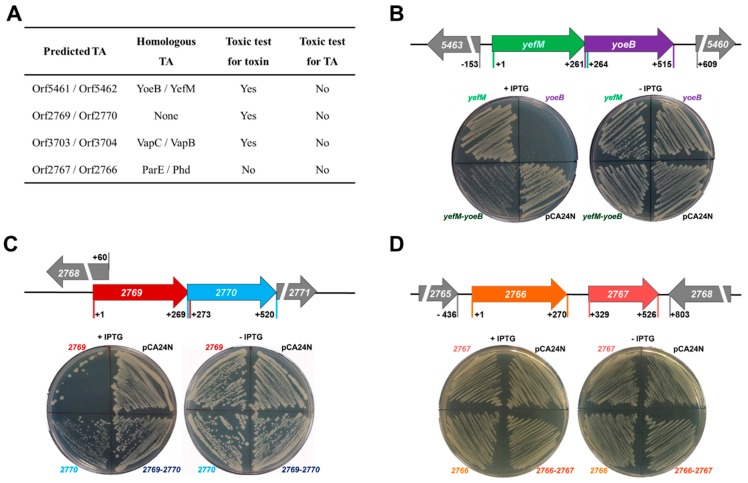
Identification of two TA Pairs. (**A**) Four potential TA loci in SCSIO 02999 genome were predicted and tested by a toxin test. *yoeB*-*yefM* (**B**), *orf2769*-*orf2770* (**C**) and *orf2767*-*orf2766* (**D**) are located in the chromosomal region of SCSIO 02999. The number indicates the relative position to the start codon of *yefM*, *orf2769* and *orf2766* (upper panel). *E. coli* K-12 BW25113 carrying the pCA24N-based plasmids were streaked on LB plates supplemented with chloramphenicol (30 µg/mL) with or without IPTG (1 mM) (lower panel). Three independent cultures were tested, and only one representative image of each strain is shown here.

Four potential TA loci (*orf3703*/*orf3704*, *orf5461*/*orf5462*, *orf2769*/*orf2770* and *orf2767*/*orf2766*) in the SCSIO 02999 genome have been predicted by the web-based tool RASTA-Bacteria [30] (Figure 1A). Among them, Orf3703/Orf3704 belong to the type-II TA VapC/VapB system [23]. The remaining three putative TAs were all investigated in this study. We first cloned the putative toxin gene and the TA operon for toxicity for these three pairs. *Orf5461* and *orf5462* encode two small proteins of 84 aa and 87 aa (Figure S1). Orf5461 belongs to the toxin YoeB family (PF06769) and Orf5462 belongs to the antitoxin YefM family (PF02604) in the Pfam database (Table S1). At the amino acid sequence level, Orf5461 shares 51% identity with YoeB in *E. coli,* and Orf5462 shares 72% identity with YefM in *E. coli* [31] (Figure S2A,B). Thus, we renamed this pair YoeB/YefM. As expected, overexpressing *yoeB* via pCA24N-*yoeB* is toxic, while coexpressing *yoeB* and *yefM* via pCA24N-*yefM*-*yoeB* significantly reduced the toxicity of YoeB in the *E. coli* K12 host (Figure 1B and Figure S3A). Another putative TA pair, *Orf2769* and *Orf2770*, encodes two small proteins of 90 aa and 83 aa (Figure S4). Overexpressing *orf2769* via pCA24N-*2769* is toxic, and overexpressing *orf2770* via pCA24N-*2770* showed slight growth inhibition. In contrast, coexpressing *orf2769* and *orf2770* via pCA24N-*2769*-*2770* is not toxic in the *E. coli* K12 host (Figure 1C and Figure S3B), suggesting that these two genes constitute a TA locus. No Pfam domains were identified in Orf2769 and Orf2770, while homologues of these two proteins were annotated as hypothetical proteins found in another *Streptomyces* sp. by blastp searching against the GenBank non-redundant database (Table S1). Noticeably, the TA pair *orf2769*/*orf2770* does not match any known TA pairs and is only found in *Streptomyces* sp. The putative TA pair *orf2767*/*orf2766* encodes two small proteins of 65 aa and 89 aa (Figure S5). Orf2766 belongs to the antitoxin Phd family (PF02604) and Orf2767 belongs to the toxin ParE family (PF05016) in the Pfam database (Table S1). Orf2767 belongs to the ParE family but shows a very low similarity (57% identity with 6% coverage) with the previously characterized type-II toxin ParE in conjugative RK2 [32] (Figure S2D). Orf2766 belongs to the Phd family but also shows a low similarity (30% identity with 51% coverage) with the type-II antitoxin Phd in bacteriophage P1 [33] (Figure S2C). However, overexpressing *orf2767* and coexpressing *orf2767* and *orf2766* are nontoxic in the *E. coli* K12 host (Figure 1D and Figure S3C). Thus, two TA pairs, YoeB/YefM and Orf2769/ Orf2770, were chosen for further study.

### 2.2. Characterization of the Type-II TA Pair YoeB/YefM

**Figure 2:**
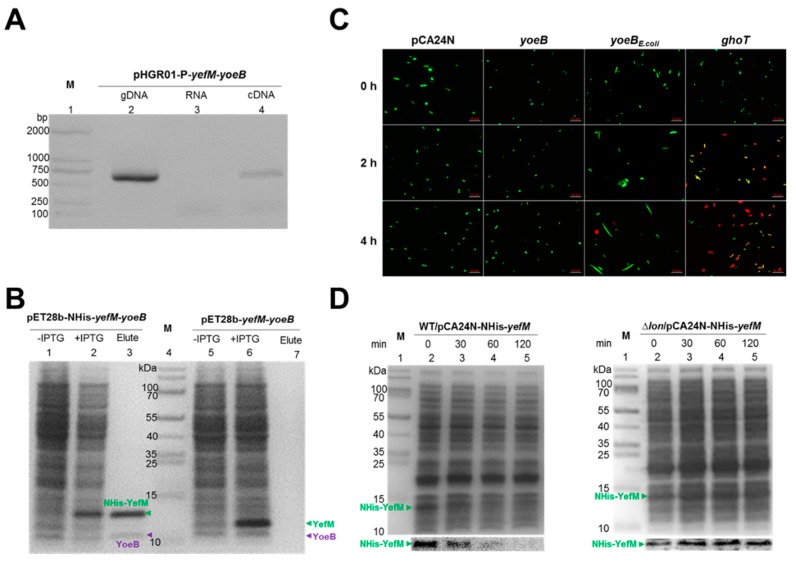
Characterization of the type-II TA pair YoeB/YefM. (**A**) Total gDNA and RNA were extracted from BW25113/pHGR01-P-*yefM*-*yoeB,* and the total RNA was used to synthesize cDNA. PCR was carried out by primer pairs using gDNA (lane 2), RNA (lane 3) and cDNA (lane 4). The DNA marker is in lane 1. (**B**) His-tagged YefM and untagged YoeB were produced from pET28b-NHis-*yefM*-*yoeB* in *E. coli* BL21. NHis-YefM (10.6 kDa) and YoeB (9.9 kDa) were induced with IPTG (1 mM). YoeB was copurified with NHis-YefM (lane 3). Cells harboring pET28b-*yefM*-*yoeB* were used as a negative control. Both untagged YefM and untagged YoeB were induced (lane 6), but neither was bound to the Ni-NTA agarose beads (lane 7). The cells without IPTG induction were used as a negative control (lane 1, 5). The protein marker is in lane 4. (**C**) Cell morphology and live/dead staining of the *E. coli* K-12 BW25113 overexpressing pCA24N, pCA24N-*yoeB*, pCA24N-*yoeB_E.coli_* and pCA24N-*ghoT* plasmids at 0 h, 2 h and 4 h. The empty plasmid pCA24N was used as the negative control and pCA24N-*ghoT* as the positive control. Three independent cultures of each strain were tested, and only representative images are shown here. (**D**) The *E. coli* K-12 BW25113 WT and ∆*lon* strains harboring the plasmid pCA24N-NHis-*yefM* were induced with 0.5 mM IPTG for 30 min, and 1% spectinomycin (100 µg/mL) was added to the strains to activate a stress response. We collected equivalent quantities of cells at 0 min, 30 min, 60 min and 120 min and then ran Tricine-SDS-PAGE (upper panel) for western blot assays (lower panel).

To test whether *yoeB* and *yefM* are cotranscribed, pHGR01-P-*yefM-yoeB* containing the 500 bp region upstream of the translational start site of *yefM* was constructed and transferred into the *E. coli* K-12 host. Total RNA was used to synthesize cDNA. The results of the RT-PCR analysis indicated the expected sizes of *yefM-yoeB,* which were consistent with the genomic DNA (gDNA) and cDNA (Figure 2A, lanes 2 and 4). No PCR products were detected in the negative controls (RNA) (Figure 2A, lane 3). These results show that the *yoeB* and *yefM* genes are cotranscribed and form a bicistronic operon. In the classical type-II TA systems, the antitoxin protein neutralizes the toxicity of the toxin through a direct protein-protein interaction [34]. To test whether YoeB binds to YefM in vivo, we performed a pull-down assay using pET28b-NHis-*yefM-yoeB* to coexpress N-terminal hexahistidine-tagged (His-tagged) YefM and an untagged YoeB toxin. Affinity purification using Ni-NTA agarose beads and subsequent Tricine-SDS-PAGE revealed that a small protein was pulled down along with the His-tagged YefM (Figure 2B, lane 1-3), and the small protein was verified by mass spectrometry to be YoeB (Table S2). We also constructed pET28b-*yefM-yoeB* to coexpress untagged YefM and untagged YoeB, and neither protein could bind to Ni-NTA beads (Figure 2B, lane 5-7). These results suggest that YoeB was purified due to its interaction with NHis-YefM. To test whether YoeB is bactericidal or bacteriostatic, we overexpressed *yoeB* and *yoeB_E.coli_* in the *E. coli* host, and live/dead staining was performed to check the viability of the cells. As shown in Figure 2C, the majority of cells overexpressing *yoeB* and *yoeB_E.coli_* remained viable after 2 h and 4 h induction with 1 mM IPTG. Moreover, when cells encounter nutritional or environmental stresses, the antitoxin of the type-II TA system is easily degraded by various intracellular proteases, such as Lon, ClpP and ClpX to free the toxins. To test which protease degrades the antitoxin YefM, the pCA24N-NHis-*yefM* plasmid was transferred into wild-type *E. coli*. When treated with spectinomycin, N-terminal His-tagged YefM was gradually degraded over time (Figure 2D). Degradation of YefM was also tested in three protease deletion mutant strains, ∆*lon*, ∆*clpP* and ∆*clpX*. As shown in Figure 2D and Figure S6, YefM remained stable only in the absence of Lon, suggesting that Lon is mainly responsible for the degradation of YefM. Collectively, these results show that YoeB and YefM constitute a *bona fide* type-II TA pair. 

### 2.3. Cross-Complementation of YoeB/YefM from E. coli and Streptomyces sp.

**Figure 3:**
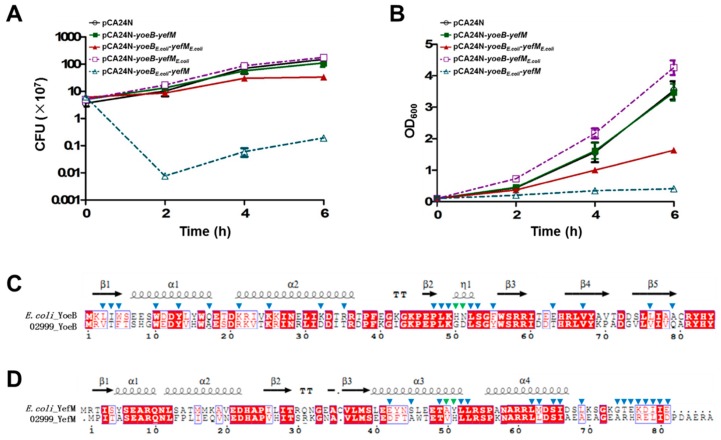
Cross-complementation of YoeB/YefM from *E. coli* and *Streptomyce*s sp. *E. coli* K-12 BW25113 harboring plasmids pCA24N-*yoeB*-*yefM*, pCA24N*-yoeB_E.coli_-yefM_E.coli_,* pCA24N-*yoeB*-*yefM_E.coli_* or pCA24N-*yoeB_E.coli_*-*yefM* were induced with IPTG (1 mM) at an OD_600_ of 0.1. (**A**) The growth of the *E. coli* strains harboring the pCA24N-based plasmids induced with IPTG (1 mM) at OD_600_ ~0.1 was monitored by absorbance at 600 nm. (**B**) At the times indicated, cell viability (CFUs/mL) was determined on LB agar plates containing chloramphenicol (30 µg/mL). Error bars indicate the standard error of the mean (*n* = 3) in A, B. (**C**) Comparison of the amino acid sequence of YoeB in *E. coli* K12 MG1655. (**D**) Comparison of the amino acid sequence of YefM in *E. coli* K12 MG1655. YefM has two protomers, and the residues of YoeB participating in direct interactions with different protomers of YefM are marked by blue triangles and green triangles [31].

Since the YoeB toxin from SCSIO 02999 is still toxic in the *E. coli* host, we next tested whether the antitoxin YefM is specific for each toxin in different bacterial hosts. Thus, we tested whether there was cross-complementation between the YoeB/YefM TA pairs from *E. coli* and from SCSIO 02999. We fused the antitoxin *yefM_E.coli_* upstream of *yoeB* to construct pCA24N-*yoeB-yefM_E.coli_* and the antitoxin *yefM* upstream of *yoeB_E.coli_* to construct pCA24N-*yoeB_E.coli_-yefM*. These plasmids were transferred into *E. coli* K-12 and were induced to overexpress the toxin and antitoxin with 1 mM IPTG. As shown in Figure 3A,B, the antitoxin from *E. coli* could counteract the toxic effect of YoeB. However, the antitoxin from SCSIO 02999 was unable to counteract the toxic effect of YoeB*_E.coli_*. Sequence analysis revealed that the key residues in YoeB and YefM that make contact with each other are different in *E. coli* and SCSIO 02999 [31] (Figure 3C,D), which may affect the direct contact between the YefM antitoxin from SCSIO 02999 and YoeB from *E. coli*. Hence, the fact that antitoxin YefM from SCSIO 02999 can only neutralize the YoeB toxin from SCSIO 02999 but not the YoeB toxin from *E. coli* suggests that the interaction between toxin and antitoxin is optimized in different bacterial hosts. 

### 2.4. Characterization of a Novel TA Pair Orf2769/Orf2770

**Figure 4:**
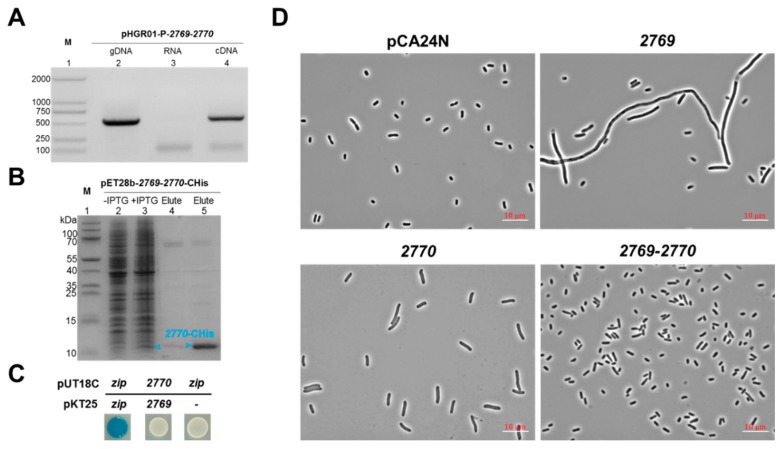
Characterization of a novel TA pair Orf2769/Orf2770. (**A**) Total gDNA and RNA were extracted from BW25113/pHGR01-P-*2769*-*2770,* and the total RNA was used to synthesize cDNA. PCR was carried out by primer pairs using gDNA (lane 2), RNA (lane 3) and cDNA (lane 4). The DNA marker is in lane 1. (**B**) His-tagged Orf2770 and untagged Orf2769 were produced from pET28b-*2769*-*2770*-CHis in an *E. coli* Rosetta host. Orf2770-CHis was induced (lane 3) and purified, while Orf2769 was unable to be copurified with Orf2770-CHis (lane 4, 5). The cells without IPTG induction were used as a negative control (lane 2). The protein marker is in lane 1. (**C**) *Orf2770* was fused to the T18 catalytic domain, and *orf2769* was fused to the T25 fragment to create an in-frame translational fusion of the T25 catalytic domain. Cells harboring pKT25-*zip* and pUT18C-*zip* plasmids were used as positive controls, and the cells harboring pKT25 (without an insert) and pUT18C-*zip* plasmids were used as negative controls. (**D**) The *E. coli* K-12 BW25113 harboring pCA24N-*2769*, pCA24N-*2770* and pCA24N-*2769*-*2770* were induced with 1 mM IPTG for 8 h, and then the cell morphology were observed under phase contrast microscope. The empty plasmid pCA24N was used as control. Three independent cultures of each strain were tested, and only representative images are shown here.

To check whether *orf2769* and *orf2770* are cotranscribed, pHGR01-P-*2769*-*2770* containing 500 bp of sequence upstream of the translational start site of *orf2769* was constructed and transferred into the *E. coli* K-12 host. Total RNA was used to synthesize cDNA. The RT-PCR analysis showed the expected sizes of *orf2769*-*orf2770,* which is consistent with the genomic DNA (gDNA) (Figure 4A, lanes 2, 4). No PCR products were detected in the negative controls (RNA) (Figure 4A, lane 3). These results revealed that the *orf2769* and *orf2770* genes are cotranscribed in the *E. coli* host and form a bicistronic operon. However, a direct interaction between Orf2769 and Orf2770 was not observed using a pull-down assay (Figure 4B) or a bacteria two-hybrid assay (Figure 4C). To test whether Orf2769 is bactericidal or bacteriostatic, we overexpressed *orf2769* in the *E. coli* host, and cell morphology was performed to check the viability of the cells. As shown in Figure 4D, the majority of cells overexpressing *orf2769* became significantly elongated after 8 h of induction with 1 mM IPTG and cells overexpressing the Orf2770 also results in slight cell elongation. Cells coexpressing Orf2769 and Orf2770 did not become significantly elongated (Figure 4D). These results are consistent with the above growth inhibition assays. Although no direct interaction between Orf2769 and Orf2770 was detected, Orf2770 is unstable, and Lon protease is mainly responsible for the degradation of Orf2770 (Figure S7). These results suggest that Orf2769 and Orf2770 may constitute a new TA pair. 

### 2.5. YoeB/YefM and Orf2769/Orf2770 Both Stabilize Plasmids in E. coli

**Figure 5:**
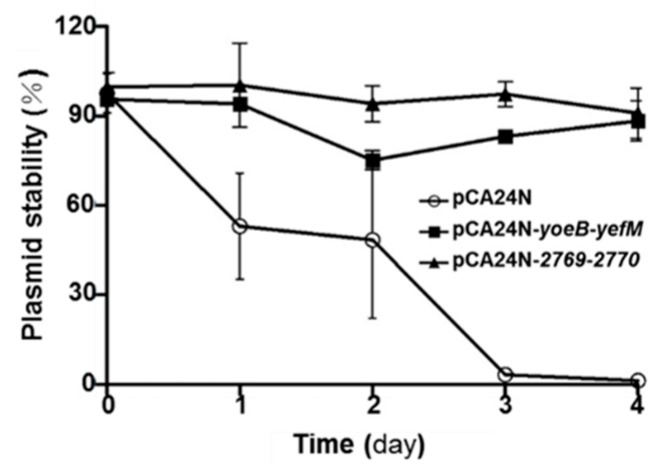
YoeB/YefM and Orf2769/Orf2770 both stabilize plasmids in *E. coli. E. coli* K-12 BW25113 harboring plasmids pCA24N, pCA24N-*yoeB*-*yefM* and pCA24N-*2769*-*2770* were used for the plasmid stability assay. The cultures were diluted 100-fold in LB medium with 0.05 mM IPTG but without any antibiotics and incubated at 37 °C every 12 h. Then, the cultures were separately plated on LB plates with or without chloramphenicol (30 µg/mL) to determine the number of CFUs every 24 h; this process was repeated for 4 days. The mean and standard deviation from three independent cultures are shown.

Both type-I and -II TA systems have the capability to increase plasmid maintenance, regardless of whether they are chromosome or plasmid origin. To test whether the two TA pairs can stabilize plasmids in *E. coli,* the high copy number plasmid pCA24N, which requires chloramphenicol to maintain its stability, was used. As expected, *E. coli* cells harboring pCA24N exhibited a higher plasmid loss rate after two days without chloramphenicol. In contrast, approximately 90% of cells harboring pCA24N-*yoeB*-*yefM* or pCA24N-*2769*-*2770* still carried the plasmids after two days (Figure 5). The results indicate that the presence of the TA pairs YoeB/YefM and Orf2769/Orf2770 from deep-sea *Streptomyce*s sp. stabilized plasmid retention in *E. coli*.

## 3. Discussion

In this study, we identified and characterized a type-II TA pair YoeB/YefM and a novel TA pair Orf2769/Orf2770 from SCSIO 02999. These results are as follows: (i) YoeB/YefM and Orf2769/Orf2770 form operons (*orf5461*/*orf5462* and *orf2769*/*2770*), and they are cotranscribed; (ii) both YoeB and Orf2769 are toxic; (iii) the cognate antitoxins YefM and Orf2770 can neutralize the toxicity of YoeB and Orf2769, respectively, and the antitoxins are unstable; (iv) YefM counteracts YoeB by direct protein-protein interaction while direct interaction between Orf2769 and Orf2770 is not observed; and (v) both YoeB/YefM and Orf2769/Orf2770 stabilize plasmids in *E. coli*. TA systems mainly spread between different microorganisms through horizontal gene transfer, and they are suggested to be closely related to the environmental adaptability of microorganisms. Our results show that although YoeB/YefM in SCSIO 02999 shares a high level of amino acid identity with YoeB/YefM in *E. coli*, the antitoxin YefM from SCSIO 02999 can neutralize the YoeB toxin from SCSIO 02999 but not the toxin from *E. coli*.; this result is consistent with previous studies, which also suggest that similar TAs have optimized interactions in their hosts, probably in response to different environmental conditions [35,36]. Our previous work demonstrated that the toxin VapC in SCSIO 02999 could cross-activate *E. coli* TA systems in a partially Lon-dependent manner. However, the qRT-PCR analysis from this study showed that neither YoeB nor 2769 could cross-activate the *E. coli* TAs (Tables S3 and S4). Collectively, these results suggest that the function and interaction of the toxin and the interaction of TAs from homologous families vary in different hosts. 

*Streptomyces* species produce more than two-thirds of the clinically useful natural antibiotics [37]. *Streptomyces* sp. is also considered to be a promising bacterial expression system for producing high levels of functional proteins [38,39]. TAs are also common genetic elements in *Streptomyces* sp.; through bioinformatics analysis, 22 putative TA systems have been identified in *Streptomyces coelicolor* A3, 27 in *Streptomyces avermitilis* MA-4680 and 14 in *Streptomyces griseus* NBRC 13350 [40]. Nevertheless, only a few TA pairs have been identified in *Streptomyces* sp. by laboratory methods [41,42]. It has been suggested that TAs YoeB/YefM from *S. lividans* is a powerful tool for producing other proteins of interest in *Streptomyces* without the use of antibiotics in the production step [43,44]. Thus, TAs are expected to be a valuable tool for the mass production of metabolites in *Streptomyces*. In this study, the TA pair Orf2769/Orf2770 in SCSIO 02999 was found not to match any known TA pairs and was only found in *Streptomyces* sp. The biochemical functions of toxins of the TA system include cleaving DNA [45,46], mRNA [47], 16S or 23S rRNA and tRNA [48,49], inhibiting gyrase activity [50] and inactivating EF-Tu [51]. Several attempts were made to purify Orf2679 toxin for in vitro functional analysis, but none was successful, mainly due to its high toxicity. Other purification methods, such as releasing antitoxin from the TA complex with denaturation, might be feasible. Nevertheless, functional elucidation of Orf2769 will be helpful to understand the physiological function of the TA pair and to their potential applications. 

## 4. Experimental Procedures

### 4.1. Bacterial Strains, Plasmids and Growth Conditions

The bacterial strains, plasmids and all primers used in this study are listed in Table 1 and Table S5. The *E. coli* strains were cultured at 37 °C in Luria-Bertani (LB) medium. As previous described, the marine-derived *Streptomyces* sp. SCSIO 02999 was isolated from a South China Sea sediment sample at a depth of 880 m [25] and the taxonomy of the strain was analyzed based on the 16S rRNA gene and was deposited in GenBank (accession No. JQ815089) [23]. Chloramphenicol (30 µg/mL) and kanamycin (50 µg/mL) were added when cells harbored plasmids with the indicated resistance gene listed in Table 1. When required, mM isopropyl-β-d-thiogalactoside (IPTG) was added as an expression inducer. 

### 4.2. Construction of Expression Plasmids

The open reading frames of *yoeB, yefM, yoeB-yefM, orf2769, orf2770*, *orf2769*-*orf2770*, *yoeB-yefM_E.coli_* and *yoeB_E.coli_-yefM* were amplified with primer pairs shown in Supplementary Table S5. Then, the purified fragments were digested with StuI restriction enzymes and ligated into the pCA24N expression plasmid by ClonExpress^TM^ II One Step Cloning Kit (Vazyme Biotech, Piscataway, NJ, USA), to generate pCA24N-*yoeB*, pCA24N-*yefM*, pCA24N-*yoeB*-*yefM*, pCA24N-*2769*, pCA24N-*2770*, pCA24N-*2769*-*2770*, pCA24N-*yoeB-yefM_E.coli_* and pCA24N-*yoeB_E.coli_-yefM*_._ The constructs were confirmed by PCR followed by DNA sequencing using primers pCA24N-f/r. The pET28b, pHGR01, pKT25 and pUT18C recombinant plasmids were constructed following similar steps. Detailed information on the primer pairs used for PCR amplification, restriction enzyme sites used in the digestion of the PCR products, and the primers used for PCR sequencing, is listed in Supplementary Table S5. 

### 4.3. Protein Expression and Purification

YefM plasmids were constructed with or without hexahistidine tagged at the *N*-terminus and YoeB was inserted into plasmid pET28b; the constructed plasmids pET28b-NHis-*yefM-yoeB* and pET28b-*yefM-yoeB* were then transformed into *E. coli* BL21. Overnight cultures of the strains were diluted with LB containing kanamycin (50 µg/mL) at an OD_600_ of 0.1, and protein expression was induced in the strains with the addition of IPTG (1 mM) when cultures reached an OD_600_ of 1.0. After inducing with IPTG for 4 h, cells were collected and resuspended in lysis buffer (50 mM potassium phosphate buffer (pH 8.0), 300 mM NaCl, and a protease inhibitor cocktail (Sigma-Aldrich, St. Louis, MO, USA)). The cells were lysed using a constant systems cell disruptor (Constant Systems Limited, Northants, UK) twice with 30 MPa. Then, the lysed cells were centrifuged at high speed (~9000 rpm) for 1 h, and the supernatant was incubated with Ni-NTA resin (Qiagen, Valencia, CA, USA) according to the manufacturer’s protocol. The protein concentration was measured by the Bi Yuntian BCA assay kit (Bioteke Corporation, Haimen, Jiangsu, China). A total of 20 μg of protein from each sample was loaded for Tricine-SDS-PAGE.

### 4.4. RNA Isolation, RT-PCR and qRT-PCR

Total RNA was isolated following the protocol of the QIAGEN RNeasy Mini Kit (Tiangen, Beijing, China). A total of 1 μg of total RNA was used to conduct reverse transcription reactions to synthesize cDNA (Promega, Madison, WI, USA). For the cotranscription assay, the gene-specific primers shown in Table S5 were used for PCR amplification. Then, 50 ng of cDNA was used to conduct qRT-PCR with the SYBR green reaction mix (Applied Biosystems Step One^TM^ Real-Time PCR System, Invitrogen, Carlsbad, CA, USA). All of the primers for qRT-PCR are listed in Table S5. The level of the *rrsG* transcript was used as a reference to normalize the gene expression data. Lower Ct (cycle threshold) values indicate higher expression levels. Fold changes in the transcripts of various targets with pCA24N, pCA24N-*2769* or pCA24N-*yoeB* were calculated as follows: log_2_((Ct target(pCA24N-*2769*)-Ct *rrsG*(pCA24N-*2769*))-((Ct target(pCA24N)-Ct *rrsG*(pCA24N)) or log_2_((Ct target(pCA24N-*yoeB*)-Ct *rrsG*(pCA24N-*yoeB*))-((Ct target(pCA24N)-Ct *rrsG*(pCA24N)).

### 4.5. Live/Dead Staining

Overnight cultures of *E. coli* K-12 BW25113 carrying the empty pCA24N, pCA24N-*yoeB*, pCA24N-*yoeB_E.coli_* and pCA24N-*ghoT* plasmids were diluted with LB with chloramphenicol (30 µg/mL) at an OD_600_ of 0.1, and IPTG (1 mM) was added at OD_600_ ~ 0.5 to induce expression of proteins. Cells were collected at 0 h, 2 h and 4 h by centrifugation at 3000 × g for 5 min, and the cells were resuspended in 300 µL of phosphate buffered saline (PBS, pH 7.4). Cells were stained with the LIVE/DEAD™ BacLight™ Bacterial Viability Kit, according to the manufacturer’s instructions. Cells were stained with 1 μL SYTO 9 nucleic acid stain (300 µL, 3.34 mM in DMSO) and 1 μL propidium iodide (300 µL, 20 mM in DMSO) and incubated in the dark for 10 min. Then, the stained cells were collected and rinsed once with PBS before resuspending in 50 µL of PBS.

### 4.6. Western Blot Analysis

The pCA24N plasmid was constructed with NHis-*yefM* or NHis-*2770* and transformed into *E. coli* K-12 BW25113 WT, ∆*lon*, ∆*clpP* and ∆*clpX* strains. The strains were incubated overnight in LB to reach exponential growth (OD_600_ ~1.0), then the cells were induced with 1% 0.5 mM IPTG for 30 min at 37°C. All cells were collected by low-speed centrifugation and resuspended in LB medium until the OD_600_ reached 1.0. The stress response was activated with 1% spectinomycin (100 µg/mL) and equal numbers of gathered cells at 0 min, 30 min, 60 min and 120 min. Protein samples were separated by Trisine-SDS-PAGE and transferred to a PVDF membrane. Western blot analysis was performed with a primary antibody raised against the His-tag and a horseradish-peroxidase-conjugated goat anti-mouse secondary antibody.

### 4.7. BACTH Assay

For BACTH assays, the coding region of *orf2770* was cloned into pUT18C, and *orf2769* was cloned into pKT25. The constructed plasmids were transformed into *E. coli* BTH101 (*cya-99*) competent cells. Cotransformed cells were plated on LB agar plates supplemented with kanamycin (50 µg/mL), ampicillin (100 µg/mL) and X-gal (20 µg/mL). The cells were cultivated at 30 °C for 24 h. The cells harboring pKT25 (without an insert) and pUT18C-*zip* (fused with a leucine zipper protein) plasmids were used as negative controls, and the cells harboring pKT25 (without an insert) and the cells harboring pKT25-*zip* (fused with a leucine zipper protein) and pUT18C-*zip* plasmids were used as positive controls [55].

### 4.8. Plasmid Stability Test

The *E. coli* K-12 BW25113 strains harboring plasmids pCA24N, pCA24N-*yoeB-yefM* and pCA24N-*2769-2770* were incubated on LB plates with chloramphenicol (30 µg/mL) at 37°C. Overnight cultures (1 mL) were washed twice with LB, resuspended in 1 mL of fresh LB and 1% of the culture reincubated with 2 mL of LB in sterile tubes. The initial CFU number of the strains was calculated and plated on LB plates and LB + chloramphenicol (30 µg/mL) plates separately. The strains were cultured at 37 °C. One percent of the culture was subcultured in sterile tubes with 0.05 mM IPTG every 12 h, and the CFU of the strains were calculated on LB plates and LB with chloramphenicol (30 µg/mL) plates every 24 h. The process was repeated for four days.

### 4.9. Sequence Alignment

Sequence analysis of the three TA pairs was performed by pfam database (http://pfam.xfam.org/search#tabview=tab0) and GenBank non-redundant database (https://blast.ncbi.nlm.nih.gov/Blast.cgi).

## Figures and Tables

**Table 1:** Bacterial strains and plasmids used in this study. Cm^R^, Km^R^ and Amp^R^ indicate chloramphenicol, kanamycin and ampicillin resistance respectively. P indicates promoter.

Bacterial Strains/Plasmids	Genotype or Description	Source
***Streptomyces* sp. SCSIO 02999**		
wild-type	A marine-derived *Streptomyces* sp., cultured in AM6 medium	[29]
***E. coli* strains**		
K-12 BW25113	*lacI*^q^*rrnB*_T14_ Δ*lacZ*_WJ16_ *hsdR*514 Δ*araBAD*_AH33_ Δ*rhaBAD*_LD78_ *rph*-*1*	[52]
K-12 BW25113∆*lon*	∆*lon* ∆*km^R^*	[52]
K-12 BW25113∆*clpP*	∆*clpP* ∆*km^R^*	[52]
K-12 BW25113∆*clpX*	∆*clpX* ∆*km^R^*	[52]
BL21(DE3)	F*^-^ompT hsdS_B_(r_B_^-^m_B_^-^) gal dcm λ*(DE3) Ω P_tacUV5_::T7 polymerase	Novagen
WM3064	*thrB*1004 *pro thi rpsL hsdS lacZ*ΔM15 RP4-1360) Δ(*araBAD*)567 Δ*dapA*1341::[*erm pir(*wt)]	W. Metcalf, UIUC
Rosetta (DE3)	F^-^ *omp*T *hsd*S_B_(r_B_^-^ m_B_^-^) *gal dcm* (DE3) *pRARE(argU, argW, ilex, glyT, leuW, proL)(*Cm^R^*)*	Novagen
BTH101	F*^-^*,cya-99,*araD139*,*galE15*,*galK16*,*rpsL1*(Str^R^),*hsdR2*,*mcrA1*,*mcrB1*	[53]
**Plasmids**		
pCA24N	Cm^R^; lacI^q^, IPTG inducible expression vector q q	[54]
pCA24N-*2769*	Cm^R^; lacI^q^, P_T5-lac_::*2769* q, PT5-lac::vapB q, PT5-lac::vapB	this study
pCA24N-*2770*	Cm^R^; lacI^q^, P_T5-lac_::*2770*	this study
pCA24N-*2769*-*2770*	Cm^R^; lacI^q^, P_T5-lac_::*2769-2770*	this study
pCA24N-*yoeB*	Cm^R^; lacI^q^, P_T5-lac_::*yoeB*	this study
pCA24N-*yefM*	Cm^R^; lacI^q^, P_T5-lac_::*yefM*	this study
pCA24N-*yoeB-yefM*	Cm^R^; lacI^q^, P_T5-lac_::*yoeB-yefM*	this study
pCA24N-*yoeB_E.coli_-yefM_E.coli_*	Cm^R^; lacI^q^, P_T5-lac_::*yoeB_E.coli_-yefM_E.coli_*	this study
pCA24N-*yoeB-yefM_E.coli_*	Cm^R^; lacI^q^, P_T5-lac_::*yoeB-yefM_E.coli_*	this study
pCA24N-*yoeB_E.coli_-yefM*	Cm^R^; lacI^q^, P_T5-lac_::*yoeB_E.coli_-yefM*	this study
pCA24N-*yoeB_E.coli_*	Cm^R^; lacI^q^, P_T5-lac_::*yoeB_E.coli_*	[54]
pCA24N-*ghoT*	Cm^R^; lacI^q^, P_T5-lac_:: *ghoT*	[54]
pHGR01	Km^R^; R6K *ori,* promoterless *lacZ* reporter vector	[55]
pHGR01-P-*2769*-*2770*	Km^R^; R6K *ori,* fused *2769*-*2770* promoter in pHGR01	this study
pHGR01-P-*yoeB-yefM*	Km^R^; R6K *ori,* fused *yoeB-yefM* promoter in pHGR01	this study
pET28b	Km^R^; *lacI*^q^, IPTG inducible expression vector	Novagen
pET28b-NHis-*yefM*-*yoeB*	Km^R^; *lacI*^q^, pET28b P*_T7-lac_*:: *yoeB-yefM* with N-terminal His-tagged	this study
pET28b-*yefM*-*yoeB*	Km^R^; *lacI*^q^, pET28b P*_T7-lac_*:: *yoeB-yefM* without His-tagged	this study
pET28b-*2769*-*2770*-CHis	Km^R^; *lacI*^q^, pET28b P*_T7-lac_*:: *2769-2770* with C-terminal His-tagged	this study
pKT25	Km^R^; encoding T25 fragment	[56]
pKT25-*2769*	Km^R^; *2769* was fused to the C termini of adenylate cyclase in pKT25	this study
pUT18C	Amp^R^; encoding T18 fragment	[56]
pUT18C-*2770*	Amp^R^; *2770* was fused to the C termini of adenylate cyclase in pUT18C	this study

## Supplementary Materials

The Supplementary Materials are available online at https://www.mdpi.com/1660-3397/17/4/211/s1.
