# Supplementary material for: Characterization of Two Toxin-Antitoxin Systems in Deep-Sea Streptomyces sp. SCSIO 02999
Source: Mar Drugs. 2019 Apr 4;17(4):211. doi: 10.3390/md17040211 (PMC6521030; doi:10.3390/md17040211)

SUPPLEMENTARY FILE

Characterization of Two Toxin-Antitoxin Systems in Deep-Sea *Streptomyces* sp. SCSIO 02999

Waner Zhan[1, 2], Jianyun Yao[1], Kaihao Tang[1], Yangmei Li[1, 2],
Yunxue Guo[1*] and Xiaoxue Wang[1, 2*]

***Correspondence:** Xiaoxue Wang; Email: xxwang@scsio.ac.cn
Yunxue Guo; Email: yunxueguo@scsio.ac.cn

Table S1. Sequence analysis results of the three putative TA pairs.

Query name	Top hit in Pfam annotation					Top hit in GenBank non-redundant database				
	**Pfam family**	**alignment start**	**alignment end**	**Description**	**E-value**	**Accession No.**	**Description**	**Query cover**	**E-value**	**Identity**
**Orf5461**	PF06769	5	84	YoeB_toxin	4.2e-41	WP_031030578	Txe/YoeB family	100%	8e-55	100.00%
**Orf5462**	PF02604	3	72	Phd/YefM_antitoxin	2.6e-18	WP_031030581	type II toxin-antitoxin system *[Streptomyces*]	100%	5e-56	100.00%
**Orf2766**	PF02604	9	57	Phd/YefM_antitoxin	4.1e-12	WP_031033384	Phd/YefM family antitoxin [*Streptomyces olivaceus*]	100%	2e-54	98.88%
**Orf2767**	PF05016	4	60	ParE_toxin	3.2e-10	WP_031033386	RelE/ParE family toxin [*Streptomyces olivaceus*]	98%	8e-36	95.31%
**Orf2769**	None					EMF54554	hypothetical protein [*Streptomyces bottropensis* ATCC 25435]	55%	1e-12	74.00%
**Orf2770**	None					WP_018845686	hypothetical protein [*Streptomyces*]	100%	8e-51	98.80%

Table S2. Mass spectroscopy results of the co-purified protein with NHis-YefM (refer to Fig. 2B, lane 3). Peptide fragments identified by mass spectrometry analysis are highlighted in different colors, and their loci in YoeB protein are also shown.

Peptide seq	Observed	Mr (expt)	Mr (calc)	ppm	M
MRVTFTSHGWEDYVHWAESDR	2609.2732	2608.2659	2608.1554	42.4	1
KVTKR	631.4585	630.4512	630.4177	53.2	2
INRLIDDITRDPFK	1716.0256	1715.0183	1714.9366	47.6	2
GIGKPEPLKGDLSGYWSR	1960.1277	1959.1204	1959.0214	50.6	1
RIDDTHR	912.5231	911.5158	911.4573	64.1	1
LVYKPADGVLVIVQARYHY	2204.3244	2203.3171	2203.2153	46.2	1

Protein YoeB [*Streptomyces* sp. SCSIO 02999]
YoeB Protein: MRVTFTSHGWEDYVHWAESDRKVTKRINRLIDDITRDPFKGIGKPEPLKGDL
Identified: MRVTFTSHGWEDYVHWAESDRKVTKRINRLIDDITRDPFKGIGKPEPLKGDL
YoeB Protein: SGYWSRRIDDTHRLVYKPADGVLVIVQARYHY
Identified: SGYWSRRIDDTHRLVYKPADGVLVIVQARYHY

Table S3. YoeB can not cross-activate *E. coli* TA systems. Fold changes of 14 TA transcripts and 1 RNase gene (*rbn*) in *E. coli* K-12 BW25113 WT or Δ*lon* cells overexpressing YoeB via pCA24N-*yoeB* as compared to empty vector pCA24N were quantified by qRT-PCR. The *yoeB* was used as positive control. Lower Ct (cycle threshold) values indicate higher expression levels. Mean and standard deviations are from three independent cultures.

Gene	WT/pCA24N	WT/pCA24N-*yoeB*		Δ*lon*/pCA24N	Δ*lon*/pCA24N-*yoeB*	
	Ct	Ct	Fold change (log_2)	Ct	Ct	Fold change (log_2)
$yoeB_{E.coli}$	27.93±1.5	27.02±1.33	0.38 ±1.8	26.54±0.1	29.44±0.95	-2.7±0.24
$yefM_{E.coli}$	26.24 ±0.46	25.31 ±0.5	1.17 ±0.52	26.4 ±0.62	27.9 ±0.99	-0.63 ±1.61
relE	26.93 ±0.18	26.79 ±0.49	0.94 ±0.38	27.25 ±0.83	27.54 ±0.73	0.48 ±1.72
relB	28.28 ±0.43	26.93 ±0.38	0.7 ±0.52	27.5 ±0.92	27.38 ±0.78	0.67 ±0.95
mazF	27.11±0.2	26.08±0.3	1.51±0.11	26.83±0.24	26.94±0.45	-0.2±0.57
mqsR	26.97 ±0.69	26.61 ±0.59	0.83 ±0.68	27.34 ±0.4	28.75 ±0.79	-1.49 ±1.03
higB	28.01 ±1.02	28.03 ±1.47	0.45 ±1.92	28.56 ±0.99	28.26 ±0.88	0.22 ±1.84
hicA	27.93 ±0.74	26.98 ±0.65	0.33 ±0.76	26.97 ±1.17	28.23 ±0.88	-0.22 ±1.67
yafO	29.42 ±1.12	28.2 ±1.44	0.86 ±1.66	28.12 ±0.73	30.87 ±0.65	-1.42 ±1.01
yhaV	29.15 ±1.09	28.62 ±1.33	0.76 ±1.46	27.33 ±0.79	29.04 ±1.1	-0.32 ±1.94
chpB	27.73 ±0.97	27.08 ±0.74	1.42 ±0.95	26.86 ±0.71	27.05 ±1.15	0.29 ±1.98
rbn	31.93 ±1.84	31.58 ±0.88	-0.21 ±1.29	29.3 ±0.17	32.38 ±0.79	-2.07 ±0.94
ralR	27.04 ±1.15	29.4 ±1.21	-2.55 ±1.64	27.36 ±0.24	30.55 ±1.68	-1.57 ±1.28
ghoT	27.46 ±0.97	29.09 ±0.21	-2.15 ±0.91	27.91 ±1.23	28.47 ±0.08	-0.21 ±1.61
yoeB	30.72 ±0.91	17.35 ±0.93	12.1 ±0.69	31.94 ±0.57	18.05 ±0.35	13.72 ±0.92
rrsG	10.95 ±0.51	10.89 ±0.31	-	11.21 ±0.48	11.67 ±0.13	-

Table S4. Orf2769 can not cross-activate *E. coli* TA systems. Fold changes of 14 TA transcripts and 1 RNase gene (*rbn*) in *E. coli* K-12 BW25113 WT or Δ*lon* cells overexpressing Orf2769 via pCA24N-*2769* as compared to empty vector pCA24N were quantified by qRT-PCR. The *orf2769* was used as positive control. Lower Ct (cycle threshold) values indicate higher expression levels. Mean and standard deviations are from three independent cultures.

**Gene**	**WT/pCA24N**	**WT/pCA24N-*2769***		**Δ*lon*/pCA24N**	**Δ*lon*/pCA24N-*2769***	
	Ct	Ct	Fold change (log_2)	Ct	Ct	Fold change (log_2)
***yoeB**$_{E.coli}$*	27.81±0.41	27.47±1.2	0.94 ±1.35	26.23±0.25	27.06±0.66	-0.69 ±0.83
***yefM**$_{E.coli}$*	26.08±0.26	26.39±0.88	0.3 ±0.99	25.56±0.68	25.99±0.36	-0.29 ±0.71
relE	27.56±0.14	27.56±0.75	0 ±0	27.33±0.18	27.38±0.63	0 ±0
relB	26.74±0.42	27.18±0.66	0.17 ±0.96	27.16±0.48	26.82±0.53	0.48 ±0.74
mazF	25.47±0.35	25.57±0.58	0.5±0.52	24.5±0.2	25.65±0.79	-1.01±0.16
mqsR	27.1±0.46	27.29±0.74	0.42 ±1	27.13±0.24	27.15±1.04	0.11 ±0.43
higB	28.28±1.01	27.46±0.65	1.43 ±1.44	27.22±0.25	27.46±1.26	-0.1 ±0.66
hicA	27.98±0.09	27.18±1.12	1.41 ±1.02	26.58±0.5	26.13±0.5	0.59 ±1.14
yafO	29.04±0.27	27.63±1.24	2.01 ±1.27	28.1±0.75	26.55±1.01	1.69 ±0.68
yhaV	28.58±0.36	26.95±1.15	2.23 ±1.09	26.47±0.66	26.16±0.93	0.45 ±0.51
chpB	27.64±0.15	27.06±1.68	1.64 ±0.52	26.98±0.62	27.15±0.76	1.18 ±0.71
rbn	29.84±0.84	28.21±1.3	1.21 ±0.28	28.57±0.4	28.76±1.32	0.86 ±0.39
ralR	29.61±0.69	27.9±1.52	0.56±0.56	28.8±0.23	28.21±1.14	0.61±0.18
ghoT	27.48±1.3	29.26±0.38	-2.38 ±1.52	27.64±0.61	26.73±0.89	1.05 ±0.77
orf2769	35.39±1.59	17.93±1.3	17.98 ±1.2	31.8±0.3	16.39±0.62	15.94 ±1.5
rrsG	10.27±0.19	10.87±0.6	-	10.87±0.36	11.01±0.49	-

Table S5. Oligonucleotides used for plasmid construction site-directed mutagenesis and DNA sequencing. If an enzymatic restriction site is included in the sequence, the enzyme restriction site is underlined. f indicates forward primer and r indicates reverse primer. P indicates promoter.

Purpose/Name	Sequence (5'-3')
**Plasmid construction**	
pCA24N-*yoeB*-f	CACCATCACCATACGGATCCGGCCCTGGTGCACCACCACCACCACAGGGTCACTTTCACGTC
pCA24N-*yoeB*-r	TAGCGGCCGCATAGGCCTCAGTAGTGGTAGCGGGCCTG
pCA24N-*yefM*-f	CACCATCACCATACGGATCCGGCCCTGATGCACCACCACCACCACCCCATCACCGCCAGCGA
pCA24N-*yefM*-r	TAGCGGCCGCATAGGCCTCACGCCCGCTCCGCGTCCGG
pCA24N-*yoeB*-*yefM*$_{E.coli}$-f	CACCATCACCATACGGATCCGGCCCTGATGCGTACAATTAGCTACAGCGAAGCGCGTCA
pCA24N-*yoeB*-*yefM*$_{E.coli}$-r	TAGCGGCCGCATAGGCCTCAGTAGTGGTAGCGGGCCTG
pCA24N-*yoeB*$_{E.coli}$-*yefM*-f	CACCATCACCATACGGATCCGGCCCTGATGCCCATCACCGCCAGCGAAGCCCGTCAGAA
pCA24N-*yoeB*$_{E.coli}$-*yefM*-r	TAGCGGCCGCATAGGCCTCAATAATGATAACGACATGC
pCA24N-*2769*-f	CACCATCACCATACGGATCCGGCCCTGATGCACCACCACCACCACGATCGGCTTCAGTGCCG
pCA24N-*2769*-r	TAGCGGCCGCATAGGCCTCATGGTGCCGATCCTACCGG
pCA24N-*2770*-f	CACCATCACCATACGGATCCGGCCCTGATGCACCACCACCACCACAGCGGTAGTAGGAAGTA
pCA24N-*2770*-r	TAGCGGCCGCATAGGCCTCAGGCGGCAGCCCCGCCGGC
pHGR01-P-*yoeB*-*yefM*-f	AGTCAATAAACCGGTGAATTCAGGTGACGAGGGCCTGCCAGT
pHGR01-P-*yoeB*-*yefM*-r	ACGACGGCCAGTGCCAAGCTTTCAGTAGTGGTAGCGGGCCTG
pHGR01-P-*2769*-*2770*-f	AGTCAATAAACCGGTGAATTCAGGCCGGCCCGCTCCACCTCG
pHGR01-P-*2769*-*2770*-r	ACGACGGCCAGTGCCAAGCTTTCAGGCGGCAGCCCCGCCGGC
pET28b-NHis-*yefM*-*yoeB*-f	TTAAGAAGGAGATATACCATGCACCACCACCACCACCCCATCACCGCCAGCGA
pET28b-*yefM*-*yoeB*-r	CGAGTGCGGCCGCAAGCTTAGTAGTGGTAGCGGGCCTG
pET28b-*yefM*-*yoeB*-f	TTAAGAAGGAGATATACCATGCCCATCACCGCCAGCGA
pET28b-*2769*-*2770*-f	TTAAGAAGGAGATATACCATGGATCGGCTTCAGTGCCG

pET28b-*2769-2770*-CHis-r	CGAGTGCGGCCGCAAGCTTAGTGGTGGTGGTGGTGGTGGGCGGCAGCCCCGCCGGCCT
pUT18C-*2770*-f	ACTCTAGAGGATCCCCGGGTACCGAGCGGTAGTAGGAAGTATTC
pUT18C-*2770*-r	ATTACTTAGTTATATCGATGAATTTCAGGCGGCAGCCCCGCCGG
pKT25-*2769*-f	CTAGAGGATCCCCGGGTACCTGATCGGCTTCAGTGCCGCCC
pKT25-*2769*-r	GAATTCTTAGTTACTTAGTCATGGTGCCGATCCTACCG
**PCR and DNA sequencing**	
pCA24N-f	GATAACAATTTCACACAGAATT
pCA24N-r	GTCAGAGGTTTTCACCGTCATCA
pET28b-f	TAATACGACTCACTATAGGG
pET28b-r	TATGCTAGTTATTGCTCAG
pHGR01-f	TTCTCCAGCCCCTCGGCGCGCATGA
pHGR01-r	CATAACCTCGCCTCCCAGGCAATGTTGGTG
pUT18C-f	GCGAGGGCTATGTCTTCTACG
pUT18C-r	GGGCTGGCTTAACTATGCGG
pKT25-f	CGCATCTGTCCAACTTCCGC
pKT25-r	CGCCAGGGTTTTCCCAGTCA
**RT-PCR**	
pET28b-*yefM-yoeB*-f	TTAAGAAGGAGATATACCATGCCCATCACCGCCAGCGA
pET28b-*yefM-yoeB*-r	CGAGTGCGGCCGCAAGCTTAGTAGTGGTAGCGGGCCTG
pET28b-*2769-2770*-f	TTAAGAAGGAGATATACCATGGATCGGCTTCAGTGCCG
pET28b-*2769-2770*-r	CGAGTGCGGCCGCAAGCTTAGGCGGCAGCCCCGCCGGCCT
**QRT-PCR**	
yoeB$_{E.coli}$-f	AGAACGCCATTTGAAGGTAAGG
yoeB$_{E.coli}$-r	TGAGCAGTGAATCGTCGGTAAC
yefM$_{E.coli}$-f	TGGAGAGGCTTGTGTTCTGATG

yefM$_{E.coli}$-r	TTTCCGTTCCTTTGCCTGAT
relE-f	CACTAAAGGAATGGCGAAAGCT
relE-r	CCAACAGAAATCACGAAAACGA
relB-f	GGTAGCATTAACCTGCGTATTG
relB-r	AGCCGTTCTTTCACTATCTCCAC
mazF-f	TATGGGCGATCTGATTTGGG
mazF-r	TTTCTTCGTTGCTCCTCTTGC
mqsR-f	CACATACACGTTTGAGTCAGGTTAA
mqsR-r	ATCAGAGTAGGTGGTCATGCTTTT
higB-f	AACATAAAACGGAGTTGGTGGC
higB-r	ACGATGAACAGCGGTAAAGAAA
hicA-f	AATCTCAGGGCGTCGATGTAG
hicA-r	CGAGTTGTTTCAGGATTGCTTTA
yafO-f	TTTCCTATAAGCGTGACGGTGTT
yafO-r	GAGGTTCAGGTTTCAGAATGGC
yhaV-f	ATCACGGTCAATCCATCATCAC
yhaV-r	GCTGAATACGGTATAGGCATCTGT
chpB-f	GTTCAAGCCTTTAATCAACTGGG
chpB-r	TAATAACGCCTCTTCCACCACC
rbn-f	AGTCGCGGCCATAGCTCTAC
rbn-r	GAAATCATTCGCCAGTTCAGTC
ralR-f	CATCAGTAACGGTGAAAGCCA
ralR-r	CCAGTGGTTCGTTTATTCCA
ghoT-f	CCTTTGTCATTATCTGGTTTATCTCAC
ghoT-r	AAAGAGAGAAAAAAGTAATGCCACAG

yoeB-f	GTGACCAAGCGGATCAACAGA
yoeB-r	CGTGACCAGTAGCCCGACA
2769-f	ATGGATCGGCTTCAGTGCC
2769-r	AATGACTGCCCCGAGGTTC
rrsG-f	TATTGCACAATGGGCGCAAG
rrsG-r	ACTTAACAAACCGCCTGCGT

Figure S1. Gene and protein sequences of *yoeB-yefM* operon in *Streptomyces* sp. SCSIO 02999. The sequences encoding *yoeB* and *yefM* is shown as indicated, and the protein sequences of YoeB and YefM were also shown together with length and size. The 500 bp of *yefM* 5' UTR region was also shown, the palindrome was highlighted, the green indicated the palindrome. The ribosome binding site (RBS) is highlighted box. The start and stop codons for *yoeB* and *yefM* are highlighted in red and blue, respectively. The overlapped four bases are underlined.

AGGTGACGAGGGCCTGCCAGTCGCCGGCGTGCCGGGCGAGGTGGTCGGCGAGCGCCTCGGAGGCGCCGAGCAC
ACCGAGGAGACGGTCGCGCAGCGGCTTGGCCGCTATCAGGGTGTCCAGCAGCTCCTGGCGGGCGGTGGGGCCG
GGCTGCGCCTCCAGGAGCCGGACCAGGCCGTGCAGGGCGAGGTCGGGGTCGGCGGTGGCGCCGAGCGCCTCCA
GGAGGACGGGGTCGGCCCGGACCTCGGCCAGCTCCGCGCCGTCCAGCAGCCGCTCGGCGGCCGAGGGATCGGT
GAAGCCGTGCCGCAGCAGTCGCGTGAAGGTACTGCTCCTGCGTCCCGGCGCCGTCATCTCGGCCTCCTGTCGG
ACCTGGGGGATTAAGGGGTACGGCTTGAGCGTAGCCGGGCAGGCGCGCGCGGGCGCCGGGACCGGTCCCGCTC
TCCGGTGGACCGCCCCGGACTCGTACGATATCTTGTACAAGCCCTGGAAGGGAGGCACCGGT

ATG CCC ATC ACC GCC AGC GAA GCC CGT CAG AAC CTG TTC CCG CTG ATA GAG CAG
GTC AAC GAG GAC CAT GCC CCG GTA CAC ATC ACC TCC CGC AAG GGA AAC GCC GTA
CTC ATG TCC GAG GAG GAC TTC ACG GCG TGG ACG GAG ACG GTG CAC CTC CTG CGC
TCG CCC AAG AAC GCC CGC CGT CTG CTC GAC TCC ATC GCG GAG GCC GAA GCG GGC
GAA GCA CGG CAT CGC GAG CTG ATC GAC CCG GAC GCG GAG CGG GCG TGA

yefM

MPITASEARQNLFPLIEQVNEDH
APVHITSRKGNAVLMSEEDFTA
WTETVHLLRSPKNARRLLDSIAE
AEAGEARHRELIDPDAERA
（**87aa, 9.79 kDa**）

GTG AGG GTC ACT TTC ACG TCC CAC GGC TGG GAG GAC TAC GTC CAC TGG GCC GAG
AGC GAC CGG AAG GTG ACC AAG CGG ATC AAC AGA CTG ATC GAC GAC ATC ACC CGT
GAC CCG TTC AAG GGC ATC GGG AAG CCG GAG CCG CTC AAG GGC GAC CTG TCG GGC
TAC TGG TCA CGG CGC ATC GAC GAC ACG CAC CGG CTC GTG TAC AAG CCC GCC GAC
GGC GTA CTG GTC ATC GTG CAG GCC CGC TAC CAC TAC TGA

yoeB

MRVTFTSHGWEDYVHWAESD
RKVTKRINRLIDDITRDPFKGIGK
PEPLKGDLSGYWSRRIDDTHRLV
YKPADGVLVIVQARYHY
（**84aa, 9.94 kDa**）

Figure S2. Comparison of amino acid sequences. (**A**) Comparison of the amino acid sequences of YoeB in *E. coli* K12 and in *Streptomyces* sp. SCSIO 02999. (**B**) Comparison of the amino acid sequences of YefM in *E. coli* K12 and in SCSIO 02999. (**C**) Comparison of the amino acid sequences of PhD in conjugative plasmid RK2 and Orf2766 in SCSIO 02999. (**D**) Comparison of the amino acid sequences of ParE in bacteriophage P1 and Orf2767 in SCSIO 02999.

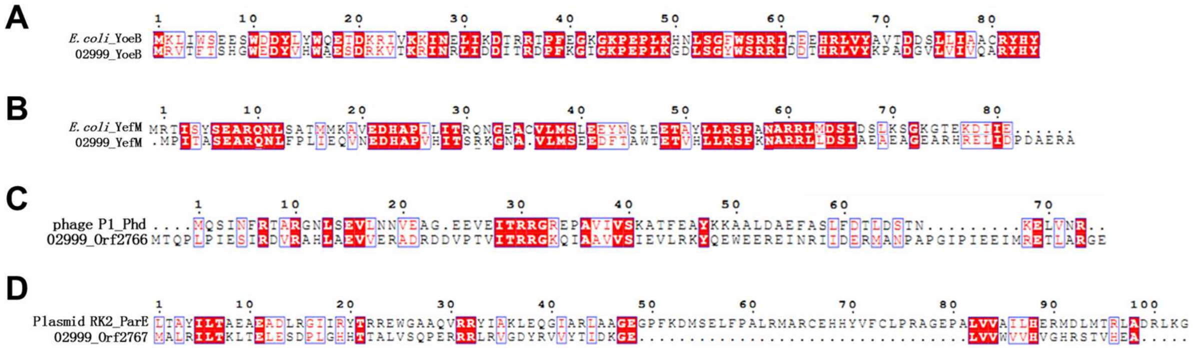

Figure S3. Growth and CFU of the three TA pairs. (**A**) Growth and CFU of *yoeB-yefM.* (**B**) Growth and CFU of *orf2769-orf2770*. (**C**) Growth and CFU of *orf2767-orf2766*. Monitor the growth of the *E. coli* strains harboring the pCA24N-based plasmids with IPTG (1 mM) at OD_{600}~0.1 by absorbance at 600 nm and cell viability (CFUs/ml) was determined on LB agar plates containing chloramphenicol (30 µg/ml) at the time points indicated.

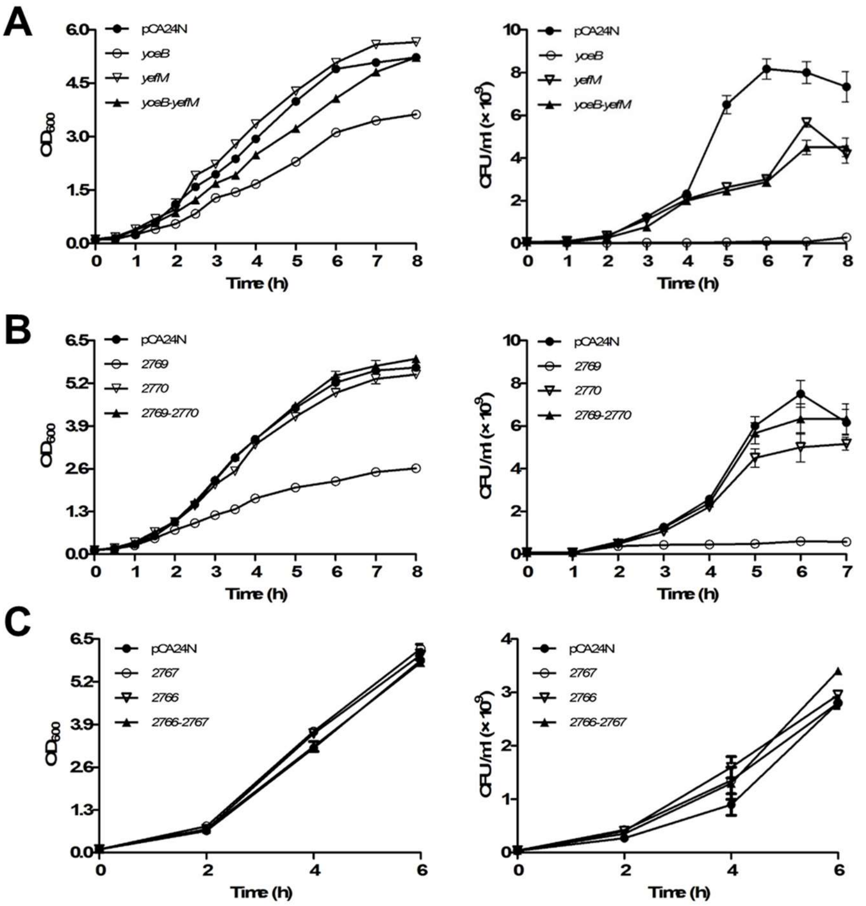

Figure S4. Gene and protein sequences of *orf2769-orf2770* operon in *Streptomyces* sp. SCSIO 02999. The sequence encoding *orf2769* and *orf2770* is shown as indicated, and the protein sequences of Orf2769 and Orf2770 were also shown together with length and size. The 500 bp of *orf2769* 5' UTR region was also shown, the palindrome was highlighted, the green indicated the palindrome. The start and stop codons for *orf2769* and *orf2770* are highlighted in red and blue, respectively. The overlapped four bases are underlined.

AGGCCGGCCCGCTCCACCTCGTCCACCAGCGACCGGGTCTCGGCGACCGCCGCCCACGCGCGAGCCC
TCTTCTCCCGGCCGGCCAGCAGCATCCGGACCCTTCCGGCGGCGGGCCCGGCGTCGGCCAACAGCGG
ACCGAGCCGTTCCGCCAGGATGGTGGCCTTGTCGACCGCACGCCGGGCGTCCGGGCCGGCCTCCACC
AGGACATGCAGGGCCATGACGAGCGCAGTGCTGCTCGGTTCCGGCCGGTCCACGTCGACGTGGACG
GCCACGGTCTCGTGCACGGCCTCGGAGAGCCGGCGGGCGGCGGCGAGCATCTGGTCGACGGTGCGC
TGCCCTACACCCGGAATCTGCCGCAGCCGGTAGGGGCCCGCTTCGAGAACACCGCCCACGGTGCGCA
GTCCGCTCCTCTCGACGCTCCCCAGCCGCAGCCGTCCTTCGGTGACGTCCTGCAGGCGGGCGACGGG
AATGGCGTCGAGCTCCCGCTTCACCGCCGCATC

ATG GAT CGG CTT CAG TGC CGC CCG TAC CGC CTC AAC TGC CCG TCC ATG GTC ACC
GAC CAC TGT CTG CGC CGC CTC GTA CAG TCG CGT TCC CCT CGC GAC TGT GTC CCG
CTC GCC ACG CCC CAC GCT TCC CGC CCC TCT CTG GCC CTC CCC GCA TCT TTC CAC
CGC CCA CAG GGG GCC TCG ACG GGC ATT CGC AGC TCC ACG CTG ATC AAC CGA ACC
TCG GGG CAG TCA TTC ACA CCC CCA GAC TTA CGA CCA CCG GTA GGA TCG GCA CCA
TGA

2769

MDRLQCRPYRLNCPSMVTDHC
LRRLVQSRSPRDCVPLATPHASR
PSLALPASFHRPQGASTGIRSSTL
INRTSGQSFTPPDLRPPVGSAP
(**90aa, 9.85 kDa**)

ATG AGC GGT AGT AGG AAG TAT TCG ATC AGC CTG CCC GAG GAT CTC GCC GAG GCC
GTA CGC GCC CAT GTC GGG CCC GGC AGT TTC TCC GCC TAC GTC GCC GAG GCT CTC
GAA CAG AGG GTC GCC ATG GAC AAG CTG CGG GAG ATC GTC GCC GAC TTC GAG ACC
GAC AAC GAA GCT CTC ACC CGC GAG GAG GTC GAG GCC GCC CGG GCG CTG CTG CGC
CAC GAC CAC CGG CAG GCC GGC GGG GCT GCC GCC TGA

2770

MSGSRKYSISLPEDLAEAVRAHV
GPGSFSAYVAEALEQRVAMDKL
REIVADFETDNEALTREEVEAAR
ALLRHDHRQAGGAAA
(**83aa, 9.01 kDa**)

Figure S5. Gene and protein sequences of *orf2767-orf2766* operon in *Streptomyces* sp. SCSIO 02999. The sequence encoding *orf2766* and *orf2767* is shown as indicated, and the protein sequences of Orf2766 and Orf2767 were also shown together with length and size. The 500 bp of *orf2766* 5' UTR region was also shown, the palindrome was highlighted, the green indicated the palindrome. The start and stop codons for *orf2766* and *orf2767* are highlighted in red and blue, respectively.

CGCGTGTAGTAGGAGGAGCTGACCCCGGCCAGCAGCGCCAGCTCCTCGCGCCGGAGCCCCGGCAC
CCGGCGCCGGTCTCCGTAGTCCGGCAGGCCGACGTCCTCCGGTCGCAGGCGGGCGCGGAGAGTCT
GGAGGAAGGCCCCGAGTTCTCTATGTCCGTGCATGTAACCCAGTATGCGAGCGCCCCCGACTCGGAG
CCTGCCCCAGCCGGGGATAGGCAAGGGAGGGCCTGGCTACCCGCCCTGAACTGCGGTGACAGCGA
TTCCCCACGTACCACGACCACGCGGTCGGCTACCCGCCCCAAGGACAGGCAGACCGTTCGCCACAC
CTCAGACCGGAATCTGCGGCACAGAACTCCCACCTGCGTAAACGCAGCACCGGAAGCGGCTATCCA
CCACCTGCCCAGCGCGTTGACGAGTCACCCGGCTCATCCAGCACCGCACAACCGCTTCACCCGGAG
CAGTTCGTGTACACATGGAACACTCGGTACACTTGAGCGC

ATG ACG CAG CCA CTG CCC ATA GAG TCC ATC CGC GAC GTA CGT GCG CAC CTG GCC
GAG GTC GTG GAG CGC GCC GAT CGG GAT GAT GTG CCC ACC GTG ATC ACT CGA CGG
GGC AAG CAG ATT GCC GCC GTC GTC TCC ATC GAG GTG CTG CGC AAG TAC CAG GAA
TGG GAA GAG CGC GAG ATC AAT CGG ATC ATC GAC GAA CGC ATG GCC AAC CCG GCA
CCC GGC ATC CCG ATC GAG GAG ATC ATG AGG GAG ACG CTG GCG CGC GGT GAG TGA

2766 → MTQPLPIESIRDVRAHLAEVVER
ADRDDVPTVITRRGKQIAAVVSI
EVLRKYQEWEEREINRIIDERMA
NPAPGIPIEEIMRETLARGE
（**89aa, 10.29 kDa**）

ATACCGAACCGTCTTCCGGCCAGAGGCACAGGCCGAGCTCCGGAAGATCCCTCGCGAC

ATG GCT CTA CGC ATC CTG ACC AAA CTG ACC GAA CTG GAG AGC GAC CCC CTC GGC
CAT CAC ACC ACC GCG CTC GTG TCC CAG CCA GAA CGC CGT CGA CTG CGG GTC GGC
GAC TAC CGG GTC GTC TAC ACG ATC GAC AAG GGG GAG CTC GTG GTG TGG GTC GTC
CAC GTC GGG CAT CGC TCC ACC GTT CAC GAA GCC TGA

2767 → MALRILTKLTELESDPLGHHTTAL
VSQPERRRLRVGDYRVVYTIDK
GELVVWVVHVGHRSTVHEA
（**65aa, 7.44 kDa**）

Figure S6. ClpXP proteases could not degrade the antitoxin YefM in *E. coli*. The mutant strains Δ*clpP* and Δ*clpX* harboring the plasmid pCA24N-NHis-*yefM* were induced with 0.5 mM IPTG for 30 min, and added 1% spectinomycin (100 μg/ml) into the strains to activate a stress response. Collected the equivalent amount cells at 0 min, 30 min, 60 min and 120 min and then ran the Tricine-SDS-PAGE for western blot assay. The Tricine-SDS-PAGE (upper panel) and western blot (lower panel) shown that the antitoxin YefM was degraded in the mutant strains Δ*clpP* (**A**) and Δ*clpX* (**B**).

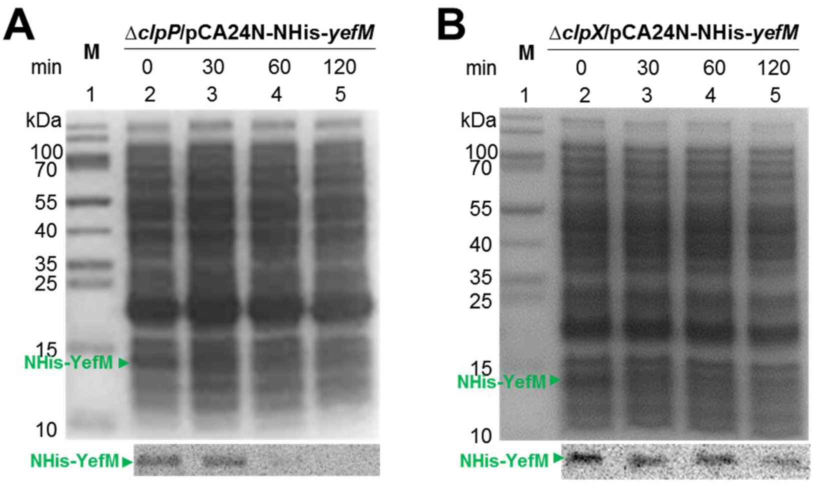

Figure S7. Lon protease degrades the antitoxin Orf2770 in *E. coli*. The strains *E. coli* K-12 BW25113 WT, Δ*lon*, Δ*clpP* and Δ*clpX* harboring the plasmid pCA24N-NHis-*2770* were induced with 0.5 mM IPTG for 30 min, and added 1% spectinomycin (100 μg/ml) into the strains to activate a stress response. Collected the equivalent amount cells at 0 min, 30 min, 60 min and 120 min and then ran the Tricine-SDS-PAGE for western blot assay. The Tricine-SDS-PAGE (upper panel) and western blot (lower panel) shown that the antitoxin Orf2770 was degraded in the WT (**A**) Δ*clpP* (**C**) and Δ*clpX* (**D**) but was not degraded in Δ*lon* strain (**B**).

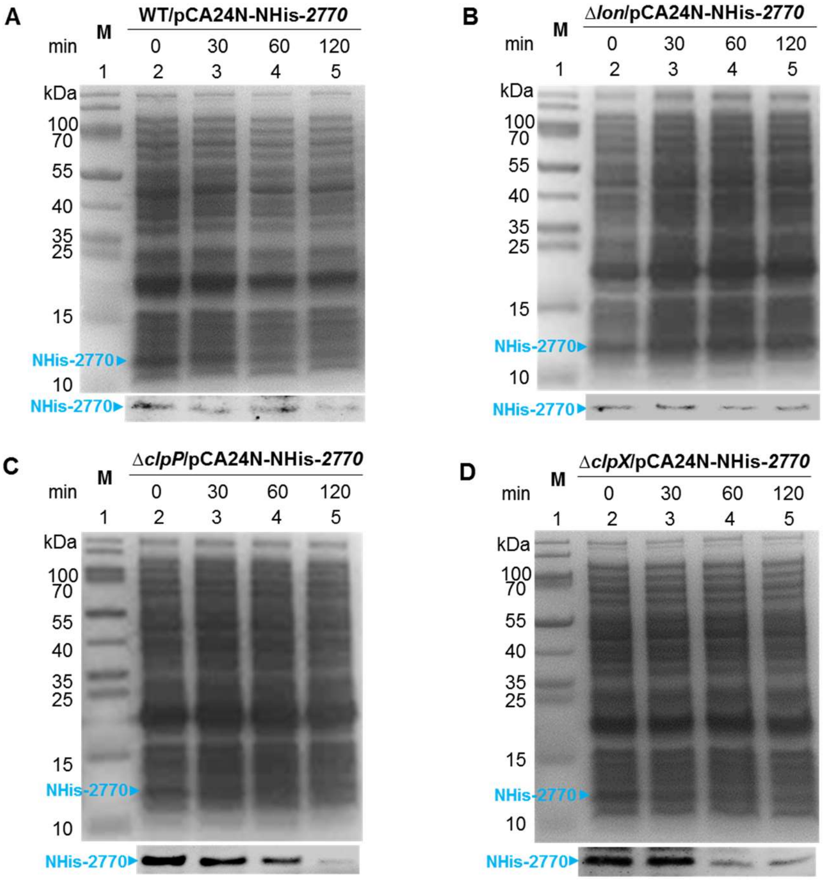

Supplement: Supplementary file 1 [file marinedrugs-17-00211-s001.pdf]
